# Evaluation of biometric formulas in the calculation of intraocular lens according to axial length and type of the lens

**DOI:** 10.1038/s41598-023-31970-5

**Published:** 2023-03-22

**Authors:** Noelia Sánchez-Liñan, Antonio Pérez-Rueda, Tesifón Parrón-Carreño, Bruno-José Nievas-Soriano, Gracia Castro-Luna

**Affiliations:** 1 Department of Nursing, Physiotherapy, and Medicine, University of Almería, Almería, Spain; 2 Department of Ophthalmology, Hospital Universitario Torrecárdenas, Almería, Spain

**Keywords:** Diseases, Health care, Medical research

## Abstract

To compare the accuracy of the modern biometric formulas in cataract surgery according to axial length and lens type. It is a Cross-sectional design from 365 patients who underwent cataract surgery. The SRK/T, Hoffer Q, Haigis, and Holladay I formulas were extracted from the IOLMaster 500 biometer. Barret formulas and the Kane were obtained from the online calculator. Patients are classified according to axial length (AL) into three groups: AL ≤ 22 mm, 22 < AL < 25 mm, and AL ≥ 25 mm. In addition, implanted intraocular lenses are classified as Monofocal, extended focus, and Multifocal. There are no significant differences between the formulas. In short, the Kane formula was more accurate than the other biometric formulas. Kane and SRK/T were the most accurate in monofocal lenses, with the lowest residual refractive error. The Holladay I formula obtained the lowest mean absolute error with the highest number of eyes with minimum residual ± 0.5Dp in the multifocal lenses in the 22 < AL < 25 mm eyes. In the long AL eyes, SRK/T and Kane's obtained the lowest mean absolute error and the best percentage of eyes with ± 0.5Dp of residual refractive error. There are no significant differences between the formulas. However Kane's formula has shown high accuracy, especially in short and long eyes with monofocal lenses.

## Introduction

Calculations of the power of the intraocular lens (IOL) to be implanted have become highly accurate since the residual refractive error is one of the important causes of dissatisfaction after cataract surgery^1^. Unfortunately, only 70% to 80% of eyes achieve postoperative refraction within ± 0.50 diopters (D) of the expected value^2^. Nowadays, biometers are based on partial coherence optical interferometry principles and can be found in many eye clinics^3^. Some of the most commonly used formulas in routine clinical practice to select the lens to be implanted are as follows:

- SRK II^4^ 2nd generation formulas that introduced the variable axial length (AL), which began to produce changes in ELP prediction;
- SRK/T^5^, Holladay1^6^ y Hoffer Q^7^ 3rd generation formulas such as Hoffer Q, Holladay 1, and SRK/T began to incorporate two variables in their calculations: AL and keratometry (K); Holladay II^8^ introduced the variables AL, corneal power, ACD, lens thickness (LT), horizontal corneal diameter (WTW), preoperative refraction and age,
- Haigis^9^ included preoperative anterior chamber depth measured from epithelium to crystalline lens (ACD), LA, and three constants dependent on the IOL**.**
- Barrett Universal II formula (BUII): is the evolution of the Barrett Universal I, the BUII is based on ray tracing (Graham Barrett, personal communication, 2019). It is available for free at https://calc.apacrs.org/barrett_universal2105/ (accesed on 15^th^ February 2023) and uses axial length (AL), keratometry (K), anterior chamber depth (ACD, measured from epithelium to lens) to predict the IOL position; LT and WTW can be entered optionally^10^.
- The Kane formula introduces the variables axial length, keratometry, anterior chamber depth, lens thickness, central corneal thickness and gender of the patient to make its calculations., the last two variables being optional^11^, whose calculations can be easily performed on the online calculator.

The correct calculation of the IOL to be implanted has importance in visual outcomes and patients' expectations. Therefore, this study aimed to investigate the final refractive results calculated by the usual biometric formulas in cataract surgery depending on different IOL formulas.

## Methods

It is a cross-sectional design based on data collected in the medical records of patients who underwent cataracts.

Sample size: Calculated with the Granmo calculator accepting an alpha risk of 0.05 and a beta risk of 0.2 in a bilateral contrast, 39 subjects in each group are required to detect a difference equal to or greater than 0.15 units. A lost-to-follow-up rate of 10% was estimated.

A database of 365 patients who underwent cataract surgery at the Oftalvist center in Almeria between January 2021 and September 2022 was created. A single eye of each patient was selected. The study complied with the declaration of Helsinki and obtained permission from the ethical committee of the Department of Nursing, Physiotherapy, and Medicine with the code EFM 179/2022. Furthermore, all patients signed an informed consent for the scientific use of their anonymized clinical data.

Inclusion criteria were: patients without previous refractive surgical procedures such as laser-assisted in situ keratomileusis (LASIK), photorefractive keratotomy (PRK), or incisional surgery, with prior corneal diseases such as keratoconus or corneal scarring, with a history of prior intraocular surgery, and with retinal abnormalities. Uncomplicated surgery.

The variables collected were:

- Demographic data such as sex and age. In addition, preoperative and postoperative corrected and uncorrected visual acuity (decimal) was obtained with the Topcon^®^ IS-600 refractive column.
- Postoperative residual refraction, preoperative, and postoperative intraocular pressure (IOP) with the Topcon^®^ TRK-2P auto refractometer, central corneal pachymetry, ant-post corneal relation, Anterior chamber depth, and white to white with PENTACAM^®^, axial length, preoperative anterior face keratometry (K1 and K2), power of the implanted lens, refractive residuals of each formula, and power of the lens calculated according to each biometric formula in emmetropia, obtained with IOL MASTER 500^®^ for the SRK/T formulas, Hoffer Q, Haigis, and Holladay 1.
- The power of the implanted lens, refractive residual, and power of the lens in emmetropia for the KANE formula were obtained using the online calculator.

Patients were classified according to axial length (AL) in three groups: short eyes (AL ≤ 22 mm) (39 eyes), normal eyes (22 < AL < 25 mm) (264 eyes), and long eyes (AL ≥ 25 mm) (62 eyes) following published e-norms methodology in ophthalmic biometry^12^. The intraocular lenses implanted are classified into three types:

- Monofocal: Asqelio^®^ monofocal (AST). Biospheric and biconvex hydrophobic acrylic lens. Optimized constant.
- Extended focus (EDOF): AcrySoft IQ Vivity^®^ (Alcon). Asymmetrical biconvex lens made of a hydrophobic acrylate/methacrylate copolymer.
- Trifocals: PhysIOL^®^ POD F (Medicalmix). The lens is made of 26% hydrophilic acrylic material with a diffractive front and aspheric back surfaces and optimized constant.

### Outcome measurements

Refractive prediction error was calculated as the difference between the spherical equivalent of the postoperative manifest refraction and the formula prediction error. A negative refractive prediction error means a myopic result, and a positive prediction error represents a hyperopic outcome. Study outcome measures included the ME and its SD, the mean absolute error, and median prediction error (MedAE) of each formula, for each group, following Wang et al.^13^ recommendations. In addition, the percentage of eyes with a prediction error within ± 0.50, ± 0.75 and ± 1.00 were also calculated.

Demographics and biometric data of patients were described with frequencies (percentages) and with a mean (SD). Data normality was assessed by the Kolmogorov–Smirnov test. Parametric one-way ANOVA and nonparametric χ2 and Kruskal–Wallis tests were applied, as appropriate, to compare demographics and biometric data from AL groups. Parametric one-sample t-test or nonparametric Wilcoxon signed-rank test (one sample) were used, as appropriate, to evaluate whether each formula's mean refractive prediction error was different from zero. The comparisons of the absolute errors were assessed using the Friedman test (nonparametric ANOVA) with Bonferroni correction, as recommended^12,14^ using the Dunn post-test. The Cochran Q test was used to compare the percentage of eyes within ± 0.5 D, ± 0.75 D, and ± 1.00 D, with Bonferroni adjustment, using Dunn post-test. A level of significance α = 0.05 was considered. Statistical analysis was performed using SPSS for Windows Software (V.27.0, SPSS).

### Ethical approval and consent to participate

This study was performed in line with the principles of the Declaration of Helsinki. Therefore, approval was granted by the Ethics Committee of the Department of Nursing, Physiotherapy and Medicine of the University of Almería Code: EFM 179/2022. Informed consent was obtained from all individual participants included in the study.

## Results

The distribution by sex was: 50.4% of patients women and 49.6% men. Table 1 shows demographic and biometric data of patients by group. The biometric data divided according to axial length shows significant differences between the depth of the anterior chamber, the white-white distance, and the power of the implanted lens. However, all of them increase with axial length.

**Table 1:** Demographic and biometric data of patients by the group.

Parameter	Group 1 (AL ≤ 22)	Group 2 (22 < AL < 25)	Group 3 (AL ≥ 25)
Age, years	77.17 ± 5.80	71.47 ± 9.1	64.54 ± 8.47
Ant-Post corneal relation	82.13 ± 1.99	82.02 ± 1.84	82.75 ± 2.20
Axial length, mm*	21.56 ± 0.39	23.31 ± 0.39	26.45 ± 1.94
Anterior chamber depth, mm*	2.72 ± 0.29	3.07 ± 0.34	3.46 ± 0.25
Mean keratometry, D*	45.96 ± 1.43	43.90 ± 1.30	43.11 ± 1.43
Corneal thickness, µm	537.87 ± 36.46	542.22 ± 38.08	527.57 ± 28.77
White to white, mm*	11.24 ± 0.25	11.71 ± 0.40	12.18 ± 0.53
Implanted IOL power, D*	26.25 ± 2.99	22.08 ± 2.17	13.64 ± 5.68

*p < 0.05 statistical significance.

Table 2 shows the predicted refractive error with standard deviation, mean absolute error, and median absolute error for each formula as a function of axial length.

**Table 2:** Refractive prediction errors, SD, MAE, and MedAE.

FORMULA	ME	SD	MAE	MedAE	± 0.50 (%)	± 0.75 (%)	± 1.00(%)
Group 1 AL ≤ 22 mm							
SRK/T	− 0.15	0.31	0.35	0.28	81.0	100.0	100
Haigis	− 0.03	0.35	0.35	0.24	71.4	95.2	100
Hoffer Q	− 0.08	0.20	0.32	0.29	81.8	95.4	100
Holladay I	− 0.20	0.23	0.30	0.26	90.5	100	100
Kane	− 0.07	0.24	0.27	0.28	90.5	100	100
Barrett	− 0.23	0.28	0.28	0.26	86.4	95.5	100
Group 2 22 < AL < 25 mm							
SRK/T	− 0.18	0.23	0.27	0.24	87.1	96.9	100
Haigis	− 0.16	0.40	0.36	0.31	75.8	93.9	100
Hoffer Q	− 0.22	0.32	0.28	0.25	87.1	97.8	100
Holladay I	− 0.23	0.25	0.26	0.22	88.9	99.1	100
Kane	− 0.17	0.28	0.28	0.24	83.0	96.9	100
Barrett	− 0.23	0.28	0.28	0.23	82.5	96.4	100
Group 3 AL ≥ 25 mm							
SRK/T	− 0.30	0.28	0.19	0.17	93.1	100	100
Haigis	− 0.43	0.36	0.27	0.24	88.9	100	100
Hoffer Q	− 0.38	0.37	0.23	0.25	93.1	96.5	100
Holladay I	− 0.37	0.37	0.22	0.17	89.7	100	100
Kane	− 0.24	0.28	0.21	0.16	92.6	100	100
Barrett	− 0.22	0.37	0.24	0.21	89.7	100	100

Percentage of Eyes Within Certain Range of Prediction Error.

*p < 0.05 statistical significance.

**Figure 1:**
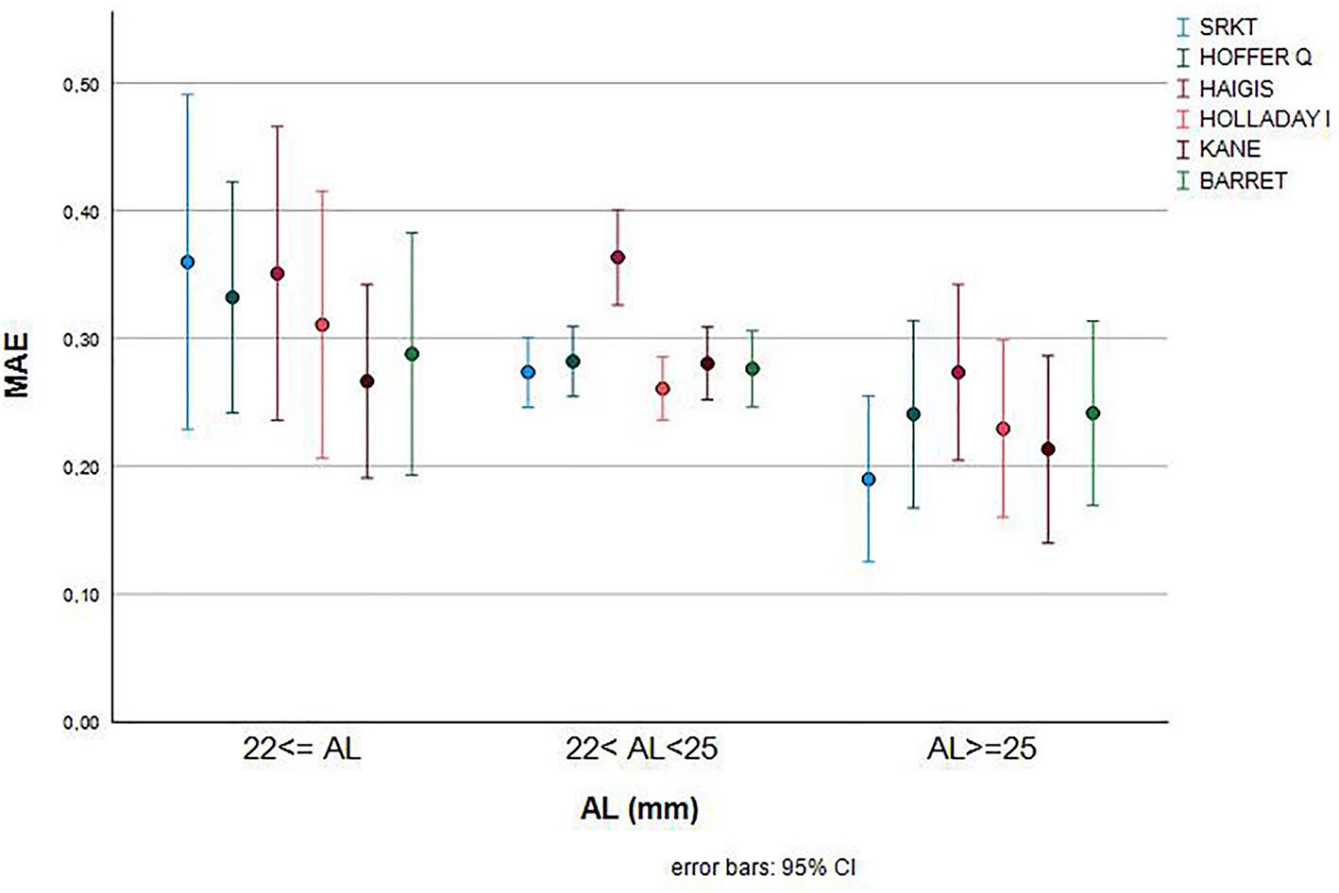
MAE according to AL and biometric formula.

The confidence interval is extensive in the extreme eyes, as shown in Fig. 1. There are no significant differences between the formulas except in the case of SRKT where there are significant differences between the extreme eyes, in short eyes (AL ≤ 22 mm), the highest mean absolute error corresponds to the Haigis and SRK/T formula. The lowest mean absolute error in short eyes was the Kane formula. Evaluating the percentage of eyes with refractive prediction errors of ± 0.5 diopters was the Holliday I and Kane formula with up to 90% of the eyes that achieved the best result.

Concerning the group of axial lengths from 22 to 25 mm, the lowest absolute error corresponded to the Holliday I formula and the highest one to the Haigis formula. The highest percentage of eyes with around 0.5 D of refractive prediction error corresponded to Holladay I.

In the long eyes (AL ≥ 25 mm), the SRK/T formula has the lowest mean absolute error, and the Haigis formula has the highest. The highest percentage of eyes with ± 0.5 D was for SRK/T formula (93.1%) (Table 2).

There are no statistically significant differences intergroup (between formulas) and intragroup (each formula according to axial length) except SRKT formula between short and long eyes (Table 3). The type and number of lenses implanted were 230 monofocal IOLs (62.90%), 63 Multifocal IOLs (17.30%) and 72 EDOF IOLs (19.80%) No EDOF lenses were implanted in eyes considered short (AL < 22), and a few EDOF lenses AL > 25 mm.

**Table 3:** Multiple comparisons.

			Mean Difference	SE	*p*-value	95% Confidence Interval	
						Lower Bound	Upper Bound
SRKT	Short AL	Normal AL	0.07	0.05	0.367	− 0.04	0.19
		LongAL	0.16*	0.06	0.023	0.02	0.30
	Normal AL	Short AL	− 0.07	0.05	0.367	− 0.19	0.04
		LongAL	0.09	0.04	0.113	− 0.01	0.19
	LongAL	Short AL	− 0.16	0.06	0.023	− 0.30	− 0.02
		Normal AL	− 0.09	0.04	0.113	− 0.19	0.01
Hoffer Q	Short AL	Normal AL	0.04	0.05	1.000	− 0.07	0.15
		LongAL	0.09	0.06	0.343	− 0.05	0.23
	Normal AL	Short AL	− 0.04	0.05	1.000	− 0.15	0.07
		LongAL	0.05	0.04	0.643	− 0.05	0.15
	LongAL	Short AL	− 0.09	0.06	0.343	− 0.23	0.05
		Normal AL	− 0.05	0.04	0.643	− 0.15	0.05
Haigis	Short AL	Normal AL	− 0.01	0.06	1.000	− 0.16	0.13
		LongAL	0.08	0.08	0.964	− 0.11	0.26
	Normal AL	Short AL	0.01	0.06	1.000	− 0.13	0.16
		LongAL	0.09	0.05	0.292	− 0.04	0.22
	LongAL	Short AL	− 0.08	0.08	0.964	− 0.26	0.11
		Normal AL	− 0.09	0.05	0.292	− 0.22	0.04
Holladay I	Short AL	Normal AL	0.04	0.04	1.000	− 0.06	0.14
		LongAL	0.08	0.05	0.366	− 0.05	0.21
	Normal AL	Short AL	− 0.04	0.04	1.000	− 0.14	0.06
		LongAL	0.04	0.04	0.738	− 0.05	0.13
	LongAL	Short AL	− 0.08	0.05	0.366	− 0.21	0.05
		Normal AL	− 0.04	0.04	0.738	− 0.13	0.05
Kane	Short AL	Normal AL	− 0.02	0.05	1.000	− 0.13	0.10
		LongAL	0.05	0.06	1.000	− 0.09	0.20
	Normal AL	Short AL	0.02	0.05	1.000	− 0.10	0.13
		LongAL	0.07	0.04	0.310	− 0.03	0.17
	LongAL	Short AL	− 0.05	0.06	1.000	− 0.20	0.09
		Normal AL	− 0.07	0.04	0.310	− 0.17	0.03
Barrett	Short AL	Normal AL	0.00	0.05	1.000	− 0.12	0.12
		LongAL	0.04	0.06	1.000	− 0.11	0.19
	Normal AL	Short AL	0.00	0.05	1.000	− 0.12	0.12
		LongAL	0.04	0.04	1.000	− 0.07	0.14
	LongAL	Short AL	− 0.04	0.06	1.000	− 0.19	0.11
		Normal AL	− 0.04	0.04	1.000	− 0.14	0.07

MAE mean difference.

*The mean difference is significant at the 0.05 level. SE: Standarized Error.

Table 4 compares the biometry formulas predictions to the lens type and the axial length. Again, there are no significant differences between the lens type and the biometry formula. However, analyzing the percentage of eyes with a refractive prediction error of ± 0.5, the Kane formula has the highest percentage of eyes with minimum refractive prediction error in short and long eyes about monofocal lenses.

**Table 4:** Percentage of eyes within certain range of prediction error by biometric formula and type of intraocular lenses.

		± 0.5 (%)	± 0.75 (%)	± 1 (%)
AL ≤ 22 mm				
SRK/T	Monofocal	84.6	100	100
	Multifocal	50.0	100	100
Haigis	Monofocal	75.0	100	100
	Multifocal	100	100	100
Hoffer Q	Monofocal	76.9	100	100
	Multifocal	100	100	100
Holladay I	Monofocal	84.6	100	100
	Multifocal	100	100	100
Kane	Monofocal	91.7	100	100
	Multifocal	100	100	100
Barrett	Monofocal	84.6	100	100
	Multifocal	100	100	100
22 < AL < 25 mm				
SRK/T	Monofocal	87.8	96.3	100
	Multifocal	90.0	97.5	100
	EDOF	81.1	94.6	100
Haigis	Monofocal	77.9	95.8	100
	Multifocal	92.1	97.4	100
	EDOF	45.4	78.7	100
Hoffer Q	Monofocal	85.0	97.9	100
	Multifocal	95.0	97.5	100
	EDOF	86.5	97.3	100
Holladay I	Monofocal	86.5	99.3	100
	Multifocal	95.1	97.5	100
	EDOF	89.2	97.3	100
Kane	Monofocal	81.6	97.2	100
	Multifocal	92.1	100	100
	EDOF	78.9	92.1	100
Barrett	Monofocal	82.8	96.6	100
	Multifocal	85.0	100	100
	EDOF	78.9	92.1	100
AL ≥ 25 mm				
SRK/T	Monofocal	89.5	100	100
	Multifocal	100	100	100
Haigis	Monofocal	94.4	100	100
	Multifocal	85.7	100	100
Hoffer Q	Monofocal	94.4	100	100
	Multifocal	100	100	100
Holladay I	Monofocal	84.2	100	100
	Multifocal	100	100	100
Kane	Monofocal	94.4	100	100
	Multifocal	85.7	100	100
Barrett	Monofocal	89.5	100	100
	Multifocal	87.5	100	100

In eyes with intermediate axial lengths (22 < AL < 25), the Holliday I best predicted the EDOF lens power than the other formulas (Table 4). No EDOF lenses have been implanted in eyes with AL ≤ 22 mm. The number of EDOF lenses implanted in long eyes (AL** ≥ **25 mm**)** is deficient, so in both categories, these lenses are not analyzed in Table 4.

## Discussion

### Group 1 AL ≤ 22 mm

Short eyes have become quite a challenge. The main problem is the high-power IOL. Moreover, a small error in the final position of the lens implies a more significant residual refractive error, which makes effectiveness much more complex end of the formulas used for the calculations^15^.

Our study shows that there are no statistically significant differences between the formulas. However there are slightly more accurate results with the Kane (MAE 0.27) and Barrett (MAE 0.28^16^. On the other hand, the highest MAE was obtained with the SRK/T and Haigis formulas (MAE 0.35)^17^.

Gavin et al.^18^, with a sample of 41 eyes, compared the Hoffer Q formula (MAE 0.78) with the SRK/T formula (MAE 0.98), obtaining worse results than ours. Arisodemou et al.^19^ compared the Hoffer Q formula with the Holladay 1 and the SRK/T in their study. This author obtained the lowest mean absolute error in AL eyes from 20.00 to 20.99 mm for Hoffer Q (MAE 0.46). Even more recent studies, such as the one conducted in 2021 by Oleksiy et al.^20^, have shown that Hoffer Q had the lowest MAE. However, in our study, the newest Kane and Barret formulas were slightly more accurate than Hoffer Q in all types of lenses.

Some studies support the efficacy of the Haigis formula in short eyes^21,22^. In these studies, the efficacy of the formulas is evaluated based on the ACD variable, with the Haigis formula being more effective in the group with ACD less than 2.40 mm compared to the Hoffer Q. In our study, the Hoffer Q was more effective than the Haigis regardless of the ACD. There are not as many articles studying the Kane formula's efficacy. Our study found similar results to the study by Darcy et al.^23^ obtaining the Kane formula was the most accurate formula in the short eyes (MAE 0.441). The Kane formula was also one of the most accurate formulas in the study by Oleksiy et al.^20^.

In conclusion, the Kane formula is reliable for performing the calculations in short eyes, with slightly better refractive results than the Hoffer Q. Kane formula is a good option for these cases of short eyes specially with monofocal lens implantation.

### Group 2 22 < AL < 25 mm

Carmona et al. had a good result with the Haigis (MAE 0.28), Holladay I, and Barrett formulas for normal eyes^24^. In the study by Connell et al.^11^ the Kane formula obtained the highest accuracy in the group of normal eyes. The same results were obtained by Darcy et al.^23^. In this study, the Kane formula was more accurate in all axial lengths. In our study, there were no ststistical differences among the formulas. However Kane was entirely accurate, but SRK/T and Holliday formulas are more effective with less MAE and more percentage of patients with a prediction error of ± 0.50.

### Group 3 AL ≥ 25 mm

Long eyes are also another fundamental challenge in the precision of the calculation of the IOL to be implanted. The results are usually residual hyperopia, regardless of whether the implanted lens has positive or negative power, although it tends to occur more with negative power lenses. For this reason, surgeons often choose residual myopia as a target in these cases^11,25^.

In our study, the recommendations of Shammas have been followed to establish the axial reference length for long eyes^12^ (more than 25 mm). However, the adjustment proposed by Wang^26^ for the Holladay 1 and SRK/T formulas has not been performed.

SRK/T (MAE 0.19) and Kane (MAE 0.21) are the most effective formulas. The highest percentage of eyes with a prediction error of ± 0.50 was obtained with the Kane formula, reaching 92.6% ^21^.

Hoffer^27^ already found that the SRK/T formula showed more accuracy with the lowest MAE in the long eyes > 24.5 mm overall (MAE 0.375) and in the mid-long subdivisions from 24.5 to 26.0 mm. (MAE 0.345) and very long > 26.0 mm (MAE 0.442).

Carmona et al.^24^ obtained a high efficacy of the Barrett formula, followed by the Kane formula (MAE 0.27). In our study, Barrett's formula showed less accuracy than the Kane formula. Aristodemu et al.^19^ also found high predictability of the SRK/T formula in eyes with AL > 26.00 mm, reaching statistical significance in AL > 27.00 mm. Jiali et al.^28^ obtained the best result in eyes with AL > 26 mm with the SRK/T formulas and the Barrett Universal and Hill-RBF formulas.

We have also observed the excellent predictability of both formulas for long eyes in the results obtained concerning the type of lens implanted. However, the Kane formula was slightly superior to SRK/T in monofocal lenses^29^.

One of the limitations of this study is that we have yet to differentiate various groups of ACD (more significant and less than 2.40 mm) for short eyes. We have used the IOL Master 500 biometer that includes keratometry measurement on the anterior surface, so the results could not be extrapolated to another biometer. It is necessary to recruit sufficient EDOF lenses to evaluate the accuracy of biometric formulas with this type of lens in short and long eyes.

## Conclusions

There are no statistically significant differences between the formulas The new generation of biometric formulas such as Kane and Barret represent a good option in all axial lengths and all types of lenses. The Kane formula achieves slightly better results especially in short and long eyes and with monofocal lenses.

## Data Availability

The dataset are available at: Castro de Luna, Gracia; Sanchez Liñan, Noelia (2023), “BIOMETRIC FORMULAS DATA”, Mendeley Data, v1http://dx.doi.org/10.17632/w47dckg3zk.1.
